# Reduced neuroprotective potential of the mesenchymal stromal cell secretome with *ex vivo* expansion, age and progressive multiple sclerosis

**DOI:** 10.1016/j.jcyt.2017.08.007

**Published:** 2018-01

**Authors:** Pamela Sarkar, Juliana Redondo, Kevin Kemp, Mark Ginty, Alastair Wilkins, Neil J. Scolding, Claire M. Rice

**Affiliations:** School of Clinical Sciences, University of Bristol, Bristol, UK

**Keywords:** cell therapy, mesenchymal stromal cells, multiple sclerosis, neuroprotection

## Abstract

**Background:**

Clinical trials using *ex vivo* expansion of autologous mesenchymal stromal cells (MSCs) are in progress for several neurological diseases including multiple sclerosis (MS). Given that environment alters MSC function, we examined whether *in vitro* expansion, increasing donor age and progressive MS affect the neuroprotective properties of the MSC secretome.

**Methods:**

Comparative analyses of neuronal survival in the presence of MSC-conditioned medium (MSCcm) isolated from control subjects (C-MSCcm) and those with MS (MS-MSCcm) were performed following (1) trophic factor withdrawal and (2) nitric oxide–induced neurotoxicity.

**Results:**

Reduced neuronal survival following trophic factor withdrawal was seen in association with increasing expansion of MSCs *in vitro* and MSC donor age. Controlling for these factors, there was an independent, negative effect of progressive MS. In nitric oxide neurotoxicity, MSCcm-mediated neuroprotection was reduced when C-MSCcm was isolated from higher-passage MSCs and was negatively associated with increasing MSC passage number and donor age. Furthermore, the neuroprotective effect of MSCcm was lost when MSCs were isolated from patients with MS.

**Discussion:**

Our findings have significant implications for MSC-based therapy in neurodegenerative conditions, particularly for autologous MSC therapy in MS. Impaired neuroprotection mediated by the MSC secretome in progressive MS may reflect reduced reparative potential of autologous MSC-based therapy in MS and it is likely that the causes must be addressed before the full potential of MSC-based therapy is realized. Additionally, we anticipate that understanding the mechanisms responsible will contribute new insights into MS pathogenesis and may also be of wider relevance to other neurodegenerative conditions.

## Introduction

Recently, there has been increasing appreciation of the potential of cell-based therapies for treatment of neurodegenerative diseases including multiple sclerosis (MS) [1]. Multipotent mesenchymal stromal cells (MSCs) have received considerable attention given that they can be relatively easily isolated from bone marrow or other tissues and expanded *in vitro*. MSCs secrete a wide range of factors and have a multiplicity of actions in diverse processes, including immunomodulation, inflammation, apoptosis and angiogenesis. Many reparative processes are now recognized to be mediated, orchestrated or stimulated by the MSC secretome–the collective term for factors secreted as soluble molecules and/or in extracellular vesicles. With respect to inflammatory demyelination, MSCs have been shown to have anti-inflammatory as well as neuro- and glioprotective effects, and administration of MSC-conditioned medium (MSCcm) improves the outcome of the MS model experimental allergic encephalomyelitis (EAE) [2]. Such properties, combined with their favorable safety profile, have accelerated translation of MSC-based therapy, which is currently being explored in clinical trials in MS [1].

Characterization of bone marrow microenvironment and sub-populations of bone marrow–derived cells such as MSCs has been relatively limited in MS [3], [4], [5], [6], [7], [8], although an increase in senescence and altered cytokine secretion have been noted [5], [6]. This is of importance and potential therapeutic relevance given that donor factors, including age, expansion *in vitro* and disease states, have previously been reported to influence MSC properties, including T-cell immunosuppression [9], with functional effects in a disease model of MS [10]. We have recently shown that the bone marrow microenvironment is abnormal in MS and that MS MSCs have reduced proliferative potential and display signs of premature aging *in vitro* [11]. However, it is not known whether MSC function is impaired in MS.

In this study, we assessed whether MSC donor age and expansion of MSCs *in vitro* alters their support for neurons under conditions of trophic factor withdrawal and whether there were differential effects of the MSC secretome depending on whether MSCs were isolated from control subjects or those with progressive MS. Furthermore, we examined whether MSC expansion and donor age or the presence of progressive MS alters neuroprotective potential of the MSC secretome using well-characterized *in vitro* assays of MSC-mediated neuroprotection [12], [13], [14], [15] in nitric oxide (NO)–induced toxicity, a mechanism known to be of pathophysiological relevance to inflammatory demyelinating disease.

## Materials and methods

### MSC isolation and culture

Bone marrow samples from control subjects who had no prior exposure to immunomodulatory drugs were obtained from the femoral shaft during total hip replacement for osteoarthritis (UK Research Ethics Committee [REC] 10/H102/69). Bone marrow from patients with progressive MS was obtained as a posterior iliac crest aspirate from participants in the trials “Assessment of Bone Marrow-Derived Cellular Therapy in Progressive Multiple Sclerosis (ACTiMuS)” (NCT01815632; REC 12/SW/0358) [16] or “Repeat Infusion of Autologous Bone Marrow Cells in MS (SIAMMS-II)” (NCT01932593; UK REC 13/SW/0255) [17].

In the full cohort, the age of control subjects (n = 9; mean age, 59.3 years) was greater than patients with MS (n = 19; mean age, 50.6 years; Student *t* test *P* = 0.004; Supplementary Table S1). There was a strong trend for duration of progressive disease to increase with age (Pearson *r* = 0.364; *P* = 0.052). Not all samples were analysed in all experiments and the number of biological replicates (n) for each experiment is presented with the results. Summary data regarding the cohort including details of exposure to disease-modifying therapies are presented as supplementary information (Supplementary Table S1). No participant with primary progressive MS (n = 8) had prior exposure to disease-modifying therapy. Of 11 participants with secondary progressive MS, five had been treated with disease-modifying therapy: two with beta-interferon, two with glatiramer and one with beta-interferon then glatiramer. No one had been exposed to disease-modifying therapy in <12 months prior to bone marrow isolation.

Control bone marrow from the femoral shaft was collected in RPMI medium (Sigma) with 1000 IU heparin. Patient samples were collected in heparin before being transported in ethylenediaminetetraacetic acid (EDTA; K2). Subsequently, marrow samples were processed identically; MSCs were isolated using a density gradient, expanded *in vitro* and demonstrated to conform to expected cell surface phenotype and mesenchymal differentiation potential [4].

### Preparation of MSCcm

Culture flasks (T175 seeded with 450,000 cells) were washed twice with Dulbecco's Modified Eagle's Medium (DMEM) to remove standard MSC culture medium. Minimum medium (MIN) consisting of 50 mL DMEM, 500 µL Pen-Strep (Gibco Penicillin-Streptomycin Ref 15140-122), 500 µL Sato concentrate (containing 100 µg/mL of bovine serum albumin, 0.06 µg/mL progesterone, 16 µg/mL putrescine, 0.04 µg/mL selenite, 0.04 µg/mL thyroxine and 0.04 µg/mL triiodothyronine) [18], 500 µL holo-transferrin (Sigma-Aldrich Ref T0665) and 250 µL L-glutamine (Sigma Aldrich Ref I5500) was added to flasks (22 mL per T175) and allowed to condition for 24 h. Conditioned medium was collected from cultures of control MSCs (C-MSCcm) or MSCs isolated from patients with MS (MS-MSCcm), centrifuged, filtered and stored at -20^o^C [14].

### Cortical neuron cultures

Isolation of rodent cortical neuron cultures was undertaken as previously described [19] and 300,000 cells/well were seeded for immunocytochemistry in a 24-well plate. For a 96-well plate, 100,000 cells/well were seeded. Incubation experiments were performed at 5 days *in vitro*.

### NO-induced toxicity

Cortical neurons were conditioned in MIN, C-MSCcm or MS-MSCcm for 3 h prior to exposure to NO (0.4 mmol/L DETANONOate for 24 h) as previously described [19].

### 3-(4, 5-dimethylthiazol-2-yl)-2, 5-diphenyltetrazolium bromide assay

Neuronal survival was quantified using the 3-(4, 5-dimethylthiazol-2-yl)-2, 5-diphenyltetrazolium bromide (MTT) assay [20]. To correct for any systematic differences between experiments, MTT signal was converted to an index; the value of MTT signal in experimental conditions was divided by that in control cultures.

### Immunocytochemistry

Immunocytochemistry with panaxonal neurofilament marker SMI312 (1:600; Covance) and neuronal marker βIII tubulin (1:600; Sigma-Aldrich) permitted determination of average axonal length per field using Image J software (1.49) as previously described [19]. Species-specific (1:500) Alexa Fluor 488- and 555-conjugated antibodies (Invitrogen) were used to visualize primary antibody staining and mounting medium with 4′,6-diamidino-2-phenylindole Vectashield for nuclear identification. The counts for the experimental conditions were divided by the value in MIN, to standardize the experiments across replicates.

### Statistical analysis

Graphs were generated using GraphPad PRISM 5 (Graph Pad Software). Statistical analysis also used GraphPad PRISM 5 other than the multiple regression model (STATA v12, StataCorp), which allowed for correlation between replicates performed at different passage number from the same individual (cluster option) where appropriate. Non-parametric bootstrap analysis was used to account for possible non-normality of the parameter's distribution. Bar graphs show mean ± standard error of the mean and regression lines were fitted with 95% confidence intervals (CIs). Values of *P *<* *0.05 were considered statistically significant.

## Results

### Reduced neuronal survival under conditions of trophic factor withdrawal with C-MSCcm isolated from late-passage MSCs

**Figure 1:**
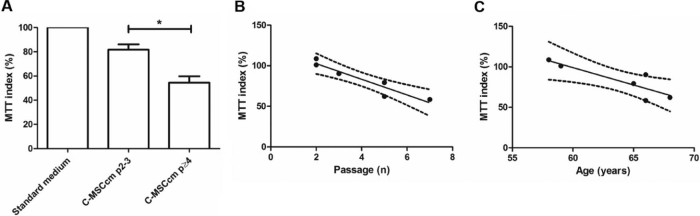
Reduced neuronal survival in the presence of C-MSCcm following trophic factor withdrawal with increasing MSC passage number (A and B) and MSC donor age (C). Cortical neuron survival was assessed using the MTT survival assay (n = 6) with results normalized to the MTT signal seen with C-MSCcm. Reduced neuronal survival under conditions of trophic factor withdrawal was observed when MSCcm was isolated from MSCs p4–7 compared with p2–3 (*P <* *0.05) and there was a negative association of neuronal survival with increasing passage number (B; Pearson *r* = −0.94; *P* = 0.005; CI, −0.99–−0.57) and age (C; Pearson *r* = −0.87; P = 0.03; CI, −0.9851–−0.1810). Not all samples were paired and where data from a single MSC culture were available at multiple passages, only a single sample was included for correlation with passage number and the earliest available passage was selected for correlation of MTT with age. A total of six biological replicates were included. There was no statistically significant correlation between age and passage number.

MSCcm has previously been shown to attenuate neuronal death associated with trophic factor withdrawal [15]. Given that *ex vivo* MSC expansion is required for therapeutic use, we examined whether neuronal survival in the presence of C-MSCcm was altered when medium was conditioned from control MSCs at early- (passage [p] ≤ 3) and late-passage number passage (p4–7). Following withdrawal of trophic factors, reduced neuronal survival as measured using MTT assay was seen with C-MSCcm isolated from late-passage MSCs (Figure 1A; *P* = 0.016). A negative correlation was seen between neuronal MTT index and passage number (Figure 1B).

### Reduced neuronal survival with C-MSCcm under conditions of trophic factor withdrawal with increasing MSC donor age

Effect of MSC donor age on C-MSCcm-mediated cortical neuron survival following trophic factor withdrawal was examined using control MSCs at p2–7. With increasing MSC donor age, neuronal survival following withdrawal of trophic factors decreased (Figure 1C).

### Reduced neuronal survival under conditions of trophic factor withdrawal with MSCcm isolated from patients with MS

We examined whether MSCcm isolated from patients with MS (MS-MSCcm) had equivalent capacity to support cortical neurons *in vitro* under conditions of trophic factor withdrawal. Effects of C-MSCcm and MS-MSCcm isolated from MSCs at p ≤ 3 (to minimize confounding effect of passage number) and at all passages examined (p2–7) on cortical neuron survival were assessed using the MTT assay as previously described. Neuronal survival following trophic factor withdrawal was reduced when MSCcm was isolated from patients with MS (MS-MSCcm) but, without correction for age and passage number, this effect did not reach statistical significance. However, using multiple regression analysis to control age and passage number, an independent effect was seen with reduced cortical neuron survival with MS-MSCcm compared with MSCcm (p ≤ 3 *P* = 0.047, CI, 0.28–38.83; p2–7 *P* = 0.049, CI, 0.1–33.88).

### Reduced C-MSCcm protection from NO-induced neurotoxicity with C-MSCcm isolated from late-passage MSCs

**Figure 2:**
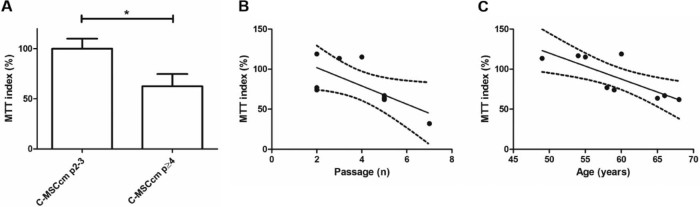
Reduced neuroprotection mediated by C-MSCcm when C-MSCcm is isolated from MSCs at late-passage number (A and B) and with increasing MSC donor age (C). Cortical neuron survival was assessed using the MTT survival assay (n = 9) with results normalized to the MTT signal seen with C-MSCcm. Reduced neuronal survival under conditions of NO-induced toxicity was observed when MSCcm was isolated from MSCs p ≥ 4 (*P <* *0.05) and MTT index decreased with passage number (B; Pearson *r* = −0.68; *P* = 0.04; CI, −0.93–−0.03). There was a negative association of neuronal survival with increasing age (C; Pearson *r* = −0.79; *P* = 0.011; CI, −0.954–−0.266). Not all samples were paired and where data from a single MSC culture were available at multiple passages, only a single sample was included for correlation with passage number and the earliest available passage was selected for correlation of MTT with age. A total of nine biological replicates were included. There was no statistically significant correlation between age and passage number.

Cortical neurons exposed to NO show decreased survival. Under these experimental conditions, MSCcm is known to be neuroprotective [19]. Using the model of NO-induced neurotoxicity, we examined neuroprotective capacity of C-MSCcm at early- (p2–3) and late-(p4–7) passage number. Neuroprotection afforded by C-MSCcm was significantly reduced when C-MSCcm was isolated from MSCs at p ≥ 4 (Figure 2A) and decreased with increasing passage number (Figure 2B).

### Reduced neuroprotection from NO-induced toxicity with isolation of C-MSCcm from older donors

Effect of MSC donor age on neuroprotection mediated by C-MSCcm in the context of NO-induced neurotoxicity was examined using control MSCs at p2–5. With increasing donor age, neuronal survival in presence of NO decreased (Figure 2C).

### Reduced neuroprotection from NO-induced toxicity with isolation of MSCcm from donors with progressive MS

In the presence of NO, decreased neuronal survival can be measured using the MTT assay but, in addition, surviving neurons have reduced axonal length, an effect reduced by MSCcm [19]. We examined whether C-MSCcm and MS-MSCcm had equivalent neuroprotective potential as assessed by MTT and relative axonal length. To minimize potential interference from effect of passage number, media conditioned from MSCs at p2–3 were examined separately although analysis was also undertaken using multiple regression and cluster analysis with all available data (p2–7).

**Figure 3:**
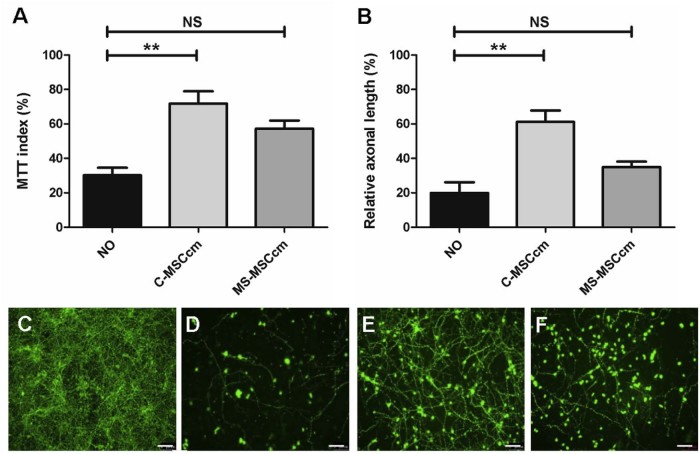
MS MSCcm fails to protect neurons from NO-induced toxicity. Neurotoxicity induced by NO was measured using the MTT survival assay (A) and quantification of relative axonal length (B–F). A neurotoxic effect of NO was observed in both assays but this was abrogated in the presence of MSCcm collected ≤p3 from control subjects (A MSCcm; MTT n = 5; axonal length n = 5) but not from patients with MS (MS MSCcm; MTT n = 19; axonal length n = 5; all with secondary progressive MS); Kruskal-Wallis with Dunn multiple comparison test was not significant (NS), **P <* *0.01. Figures C–F show representative images of cortical neurons stained with SMI-312 (green) in MIN (C), in MIN when exposed to NO (D), in the presence of NO and C-MSCcm (E) and NO with MS MSCcm (F). Scale bar = 100 µm.

Previously reported neuroprotective effects of MSCcm were replicated when conditioned medium was collected from MSC cultures (≤p3) isolated from control subjects; significant reduction in neuronal survival and relative axonal length were no longer observed when neurons were exposed to NO and C-MSCcm (Figure 3). However, when conditioned medium was harvested from MSCs (≤p3) collected from patients with progressive MS (MS-MSCcm), only a trend toward a neuroprotective effect was seen (Figure 3). Using the regression model to account for age and passage number (p2–3), an independent, negative effect of progressive MS was observed in the MTT assay (*P* = 0.04; CI, 0.90–38.62) and measurement of relative axonal length (*P *<* *0.001; CI, 19.56–66.58).

Accounting for age, passage number (p2–7) and repeated sampling from the same individual using multiple regression with cluster option, neuroprotective effect of C-MSCcm and MS-MSCcm under conditions of NO toxicity was examined using conditioned medium collected from all available samples. An independent, negative effect of presence of progressive MS was again seen on neuroprotective potential of MSC secretome in both MTT assay (C-MSCcm n = 9; MS-MSCcm n = 19; *P* = 0.043; CI, 0.56–34.3; total number of observations, 36) and in assessment of relative axonal length (C-MSCcm n = 5, MS-MSCcm n = 6; *P* = 0.032; CI, 1.84–42.17; total number of observations, 16). No differences were observed between primary and secondary progressive cohorts, duration of progressive disease or with prior exposure to MS disease-modifying therapies.

To exclude the possibility that an excessively high concentration of trophic factors in MS-MSCcm was toxic, the MTT model was used to explore neuroprotective effect of reduced concentrations of MS-MSCcm. However, the maximum effect was seen with 100% MS-MSCcm (data not shown).

## Discussion

We have demonstrated that improved neuronal survival mediated by MSC secretome under conditions of trophic factor withdrawal reduces with *ex vivo* expansion of MSCs and with increasing MSC donor age. We have also identified that, when MSCs are isolated from patients with MS, capacity of MSC secretome to support neuronal survival under conditions of trophic factor withdrawal is reduced. Furthermore, using NO-induced neurotoxicity, we have shown that neuroprotective potential of MSC secretome decreases with donor age and with *ex vivo* expansion of MSCs and that there is an independent negative effect on neuroprotective potential of the MSC secretome when the MSC donor has progressive MS.

The multi-faceted reparative potential of MSCs, including, for example, immunomodulatory and neuroprotective properties, makes MSCs attractive candidates for cell-based therapy in neurological conditions. Indeed, clinical trials using expansion of autologous MSCs are now in progress for several neurological diseases, including MS [1], [21], [22]. Furthermore, the increasing recognition of the importance of paracrine factors in MSC-mediated repair and regeneration has focused considerable attention on the MSC secretome, and the possibility of developing cell-free therapeutic interventions has been raised [23], [24]. Indeed, the importance of the secretome has been highlighted by its inclusion in the International Society for Cellular Therapy's recommendations regarding functional assessment of MSCs [25].

Increasingly, however, it is recognized that bone marrow microenvironment and function of cells of the stromal compartment are affected by disease states not previously thought to be primarily associated with marrow pathology [26], [27], [28], [29]. This contrasts with studies where “priming” by pre-exposure of MSCs to noxious stimuli was associated with increased effectiveness [30], and suggests that, under prolonged exposure to stressors as may occur in chronic disease, putative disease-ameliorating responses of MSCs may not be maintained. Alternatively or additionally, MSCs themselves may be directly targeted by the disease process.

In addition to disease-specific effects, deleterious effects of aging and *in vitro* proliferation on MSC-based therapy are now also recognized as factors that may limit effectiveness of MSC-based therapy [11], [31], particularly if autologous cells are used. Both age and *in vitro* expansion induce a variety of structural and functional changes in MSCs [32], [33], [34], including alterations in cytoskeleton and reduced capacity for migration and homing [35], [36], [37], [38], impaired hematopoiesis [39], reduced immunosuppressive potential [40] and reduced MSC-mediated anti-proliferative effects [41], [42]. With particular relevance to demyelinating disease, aging has been reported to have deleterious effects on beneficial impact of adipose-derived stem cells in EAE [10].

We used a multiple regression model to account for differences in age between control and MS cohorts, although becasuse the MS cohort was younger we are more likely to have under-estimated disease-related effects. Aside from age-mismatch between cohorts, an additional potential limitation of our study is difference in MSC origin between patients with MS and control subjects. Notably, however, pelvic marrow is generally accepted as the gold standard for isolation of MSCs [43]. That the control cohort had osteoarthritis could be a possible additional confounding effect, although, aside from age-related effects, there have been no consistent reports of the effects of osteoarthritis on isolation and proliferation of MSCs from femoral shaft marrow [44], [45], [46], [47].

Although none of the control subjects were exposed to immunomodulatory drugs, they may have had anti-inflammatory medications, such as non-steroidal anti-inflammatory drugs (NSAIDs). The effect of such drugs on MSC-mediated neuroprotection has not, to the best of our knowledge, been examined. Alterations of MSC gene expression by analgesics and anti-inflammatory drugs have been noted *in vitro* [48], and, in general, deleterious effects of anti-inflammatory medications on MSC function have been documented including anti-proliferative effects [49], [50], impaired migration [50] and induction of apoptosis [51]. The effects of these medications *in vivo* is more difficult to determine but compensatory mechanisms have been noted in canine MSCs *in vivo* [52]. There is certainly no suggestion that NSAIDS are likely to improve the reparative function of MSCs and, overall, we consider it unlikely that medication-related effects underlie our findings.

Although a minority of patients with MS were exposed to disease-modifying therapy, there was no difference in neuroprotective potential of MSC secretome when cells were isolated from subjects with primary versus secondary MS or variation by exposure to disease-modifying therapy. We cannot comment on whether MSCs from patients with relapsing-remitting MS would have an altered secretome compared with control subject who do not have MS because we have had no access to bone marrow from these patients.

Although there are data to support the use of xenogeneic models in assays of MSC-mediated immunosuppression, we acknowledge that *in vitro* experiments using cells of different species may not accurately mimic paracrine function *in vivo* [53]. Nonetheless, we consider that our finding of reduced neuroprotective potential of the MS-MSC secretome requires further investigation, including an exploration of mechanism(s) involved. Given that application of NO causes neuronal cell death and axon loss via complex mechanisms, including formation of reactive nitrogen species, inhibition of mitochondrial respiration [54] and excitotoxicity [55], we anticipate that MS-MSCs will secrete reduced levels of anti-oxidants as well as altered levels of cytokines and growth factors and may have impaired mitochondrial and peroxisome function, all of which have been implicated in MS pathophysiology. We recommend further analyses to examine whether MSCcm isolated from subjects with MS has other functional deficits of relevance to protective and reparative potential including but not limited to anti-inflammatory and immunomodulatory effects.

For optimization of MSC-based therapy in MS, careful quality control of donor MSCs will be required and our studies support the recommendation that this includes assessment of MSC secretome [25]. We anticipate that understanding mechanisms responsible for reduced neuroprotection afforded by MS secretome in MS will yield new insights into MS pathogenesis with potential for development of biomarkers of disease activity and prognosis as well as identifying novel treatments either via optimization of cell-based therapy or using small molecules.

## Appendix Supplementary material

Table S1 Cohort characteristics. Table S1
