# Supplementary material for: Reduced neuroprotective potential of the mesenchymal stromal cell secretome with ex vivo expansion, age and progressive multiple sclerosis
Source: Cytotherapy. 2018 Jan;20(1):21–8. doi: 10.1016/j.jcyt.2017.08.007 (PMC5758344; doi:10.1016/j.jcyt.2017.08.007)
Supplement: Table S1 — Cohort characteristics. [file mmc1.docx]

Supplementary Table 1. Cohort characteristics.

| Subject | Age (y) | Gender | Classification of MS | Duration of disease progression (y) | Prior disease-modifying therapy |
| --- | --- | --- | --- | --- | --- |
| Control 1 | 54 |  | N/A | N/A | N/A |
| Control 2 | 60 |  | N/A | N/A | N/A |
| Control 3 | 49 |  | N/A | N/A | N/A |
| Control 4 | 58 |  | N/A | N/A | N/A |
| Control 5 | 59 |  | N/A | N/A | N/A |
| Control 6 | 66 |  | N/A | N/A | N/A |
| Control 7 | 65 |  | N/A | N/A | N/A |
| Control 8 | 55 |  | N/A | N/A | N/A |
| Control 9 | 68 |  | N/A | N/A | N/A |
| Mean (y) | 59.3 |  |  |  |  |
| Median (y) | 59 |  |  |  |  |
|  |  |  |  |  |  |
| MS 1 | 33 | M | SP | 3 | Beta-interferon |
| MS 2 | 50 | F | SP | 7 | Beta-interferon |
| MS 3 | 50 | M | SP | 5 | None |
| MS 4 | 59 | F | SP | 15 | None |
| MS 5 | 55 | M | SP | 2 | None |
| MS 6 | 57 | F | SP | 3 | Glatiramer |
| MS 7 | 41 | F | SP | 2 | Glatiramer |
| MS 8 | 58 | F | PP | 10 | None |
| MS 9 | 50 | F | PP | 4 | None |
| MS 10 | 48 | F | PP | 15 | None |
| MS 11 | 48 | M | PP | 4 | None |
| MS 12 | 47 | F | PP | 6 | None |
| MS 13 | 49 | M | PP | 2 | None |
| MS 14 | 64 | M | PP | 15 | None |
| MS 15 | 49 | F | PP | 14 | None |
| MS 16 | 53 | F | SP | 3 | None |
| MS 17 | 47 | F | SP | 14 | None |
| MS 18 | 57 | M | SP | 11 | None |
| MS 19 | 47 | F | SP | 9 | Beta-interferon, Glatiramer |
| Mean (y) | 50.6 |  |  | 7.6 |  |
| Median (y) | 50 |  |  | 6 |  |

Summary data for both control and MS cohorts, including information on MS classification and prior exposure to disease-modifying therapy.

M, male; F, female; N/A, not available; SP, ; PP, .
